# Microbial Musings – February 2021

**DOI:** 10.1099/mic.0.001046

**Published:** 2021-02-26

**Authors:** Gavin H. Thomas

**Affiliations:** ^1^​ Department of Biology, University of York, York YO10 5YW, UK

The big microbiology story this month is the renewed search for Martian microbes, with the successful landing of NASA’s Perseverance rover on 18 February, after its 7 month journey from Earth. By landing in the Jezero Crater, which was once thought to have been full of water, the scientists are hoping to collect a series of soil and rock core samples for analysis back on Earth for signs of life. We should not hold our breath, as we will have to wait for another rover to go and collect them and return them to Earth, but this is still an important and celebrated event in humanity’s search for life on the Red planet.

We start with the latest paper from the Whitchurch lab (@Cwhitch) at the Quadram Institute, Norwich, UK, now becoming a regular feature of the *Microbial Musings*. The new paper is a continuation of their ‘explosive cell lysis’ hypothesis, as a mechanism for the release of glue-like extracellular DNA (eDNA) by *Pseudomonas aeruginosa *, which is involved in the formation of biofilms by this important pathogen [1]. The explosive bit of this process is mediated by a peptidoglycan degrading endolysin called Lys, which, when exported, weakens the cell wall of the producing cell and surrounding cells to the point where they rupture due to osmotic pressure, a mechanism reminiscent of the action of β-lactamase antibiotics. In this important new study Amelia Hynen from the iThree Institute (@ithreeinst) at the University of Technology, Sydney, Australia, working with Sydney and Norwich colleagues James Lazenby (@ScienceBear3), George Savva (@georgemsavva), Laura McCaughey (@LauraCMcCaughey), Lynne Turnbull (@lynnet3) and Laura Nolan (@LauraNolanLab), elucidate how the Lys protein gets out of the producing cell and more about the process of eDNA release in early biofilm formation [2]. Phage endolysins, which is what Lys is, are normally secreted across the inner membrane by holin proteins, and in fact the gene encoding Lys is adjacent to one, *hol*, that is an obvious candidate. However, *P. aeruginosa* PAO1 has two other holin-encoding genes, which are also tested in this study. Using plate- and liquid-based biofilm assays, the authors demonstrate clearly that *hol* is not the only holin that can result in export of Lys, while a triple deletion of all three completely abolished eDNA formation. While the Hol protein appears to be the most important, the roles of the other two vary depending on the conditions, but nonetheless the data suggests export of Lys by non-cognate holins. Using their strains with different holin activities they then investigate their function during early biofilm formation and find that holin-mediated eDNA release is important for the initiation of microcolonies during early states of liquid (submerged) biofilm formation and that this phenotype is required for grow of these into larger microcolonies [2]. These data are also very interesting as it is already known that the laying down of another component of the *P. aeruginosa* biofilm matrix, the polysaccharide called Psl, is important for the initiation of biofilm formation [3] and the authors argue that their data is consistent with eDNA production occurring before this process, which then interact with each other to form the biofilm skeleton [4].

Sticking with the Pseudomonads, our next paper is about the uptake and reduction of the environmentally toxic tellurium oxyanion, tellurite. The study of bacteria that can remove tellurite from the environment has revealed that they can either modify it to a gaseous dimethyl telluride form or reduce it fully to elemental tellurium [5]. The soil bacterium *Pseudomonas putida* KT2440 is known to use the later route to detoxify tellurite, forming a black precipitate inside the cells. In this paper Rafael Montenegro (@RafaMM19), Sofía Vieto (@sofivieto) and colleagues from the group of Max Chavarría (@MaxChavarriaV), at the Centro Nacional de Innovaciones Biotecnológicas (CENIBiot), Costa Rica, have investigated how the tellurite enters the cell and identified one of the transporters involved [6]. They studied the *P. putida* orthologue of an inorganic phosphate transporter, PitB, as the *Escherichia coli* PitA protein was known to be involved in tellurite uptake in *E. coli* [7]. First they constructed a Δ*pitB* strain using a CRISPR/Cas9 method and find that the resulting strain is more resistant to tellurite than the parent strain, with clear phenotypes in liquid and solid media. Despite this, the bacteria still produce cytoplasmic tellurium nanostructures, so it is not that no tellurite is entering the cell, rather it seems likely that the reduced rates of uptake allows the cells to detoxify it fast enough to not impact so greatly on growth. The authors aim to discover further transporters involved in tellurite uptake as part of their long-term aim to develop *P. putida* as a biotechnological solution for bioremediation of environmental tellurite.

Following from the characterization of one particular transporter, we switch to a methods paper from the group of Douglas Kell (@dbkell) at the University of Liverpool, UK, who has expanded a method he has been using in his lab to attempt to characterize the function of many predicted transporter proteins [8, 9]. The study exploits the fact that different fluorescence dyes accumulate to different degrees in bacteria through a balance of uptake and efflux, methods that have been used before with individual dyes to study efflux pumps [10]. In their study, which used *E. coli* as a test system, these fingerprints are used for a set of dyes, which gave the best performance in a flow cytometer and are screened against a large set of strains from the KO library of single-gene knockout [11]. The results are complex but clear patterns emerge for many transporters of unknown function when viewed across the library and compared to the wild-type strain. Some known mutants are included as controls, including one lacking the well-known *tolC* gene, which encodes an important outer membrane protein that couples with different RND efflux pumps [12]. This strain has intrinsically higher levels of fluorescent for many of the dyes, consistent with the loss of its promiscuous efflux function, but also some dyes accumulate less than the wild-type strain suggesting other more complex phenotypes. In contrast there are other genes of unknown function, such as *yihN*, which compared to the wild-type gave consistently lower levels of accumulation of almost all the dyes, suggesting some generic function in uptake of dyes or a more general control of membrane permeability as the fluorophores are structurally highly heterogeneous. The method looks most suited in the hunt for new efflux proteins and could be applied to many different bacteria through use of different subsets of the dye library. While there are some caveats to its application, which the authors acknowledge openly, the almost total lack of any other high-throughput methods to screen for transporters of unknown function makes this an interesting and accessible route to generate hypotheses for further experimental characterisation.

The next two papers both draw on aspects of how core cellular information transfer systems, namely RNA polymerase and the ribosome, are regulated by nutrient starvation. Starting with the ribosome, this issue contains a fascinating review of the process of ribosome hibernation [13]. During nutrient stress, specifically the stringent response, the cellular growth rate drops, and transcription of rRNA operons is heavily downregulated. However, what happens to all the ribosomes that already exist, which would burn large amounts of energy if not controlled? The review, by Anil Ojha and colleagues from the Wadsworth Center, New York, USA, describes a number of systems that allow bacteria to store away their ribosomes for a rainy day through the binding of other cellular proteins that stabilize and inactivate them. After describing these processes across bacteria, the authors focus specifically on the importance of this process in *Mycobacterium tuberculosis *, known to microbiologists for being able to slow its growth to almost zero and persist in the human body for many years. The authors outline the evidence that ribosome hibernation is an important part of this adaptation and argue that zinc depletion is a key signal for this process to initiate. Clearly improved understanding of this process and how it enables *M. tuberculosis* to persist so effectively could lead to new routes to target these bacteria when in this state and improve treatments for this chronic infection of humans.

The second paper on this looks at DksA, a transcription factor that appears to potentiate the effect of the stringent response effector molecule ppGpp to inhibit transcriptional elongation by RNA polymerase, particularly to regulate expression of rRNA operons in *E. coli* [14, 15]. It is also known to have additional effects and in the related γ-proteobacterium *Vibrio cholerae* regulates virulence-factor production, including cholera toxin (CT), work published from Rupak Bhadra’s group from the Indian Institute of Chemical Biology in India [16]. In this new paper from Madeline Sofia and Michelle Dziejman, from the University of Rochester, USA, the authors examine these additional functions in clinical strains lacking CT [17]. These strains use a type-three secretion system to deliver key virulence factors, and the production of these is not altered in the Δ*dksA* strain, which also lacks any *in vivo* colonization defect. However, the authors find that the *dksA* gene is induced by bile and that the mutant produced weaker biofilms than the parent strain and also had altered motility. Together their data suggest that the *V. cholerae* DksA is integrated into complex regulatory networks outside of its ‘typical’ role in the stringent response.

Stuart Cantlay and colleagues working the lab of Joseph McCormick at Duquesne University, Pittsburgh, USA, have now followed on from their interesting 2017 study that proposed a mechanism for FtsZ independent cell division in the Actinobacterium *Streptomyces venezuelae* [18], work which was done with the group of Jeff Errington FRS (@Errington_Lab) in Newcastle, UK. FtsZ is usually an essential protein in bacteria, as it plays a key role in forming the divisome required for cell division, but for *Streptomyces* it is actually dispensable for vegetative cell growth, which occurs at the cell tip, a growth mode more commonly associated with filamentous fungi [19]. However, FtsZ is still essential for spore formation where the divisome must form. In the new study, Cantlay has looked more at the function of the FtsZ protein in this bacterium, which is amenable to live-cell imaging as it can differentiate under submerged conditions [20]. They show that removal of most of the other core proteins of the divisome only result in partially blocks in sporulation septum formation. In these strains the FtsZ ring assembles, but then appears to be unstable, which might partially explain these phenotypes. The authors note that similar phenotypes are seen after disruption of the newly identified dynamin-like proteins [21] and that much more work is needed to fully understand how they work together to coordinate this process in this important antibiotic producing bacteria.

This month our Editor’s choice has been chosen by senior editor Tracy Palmer FRS and is the second paper in this issue from authors at the iThree Institute (@ithreeinst), University of Technology, Sydney, Australia. This paper from Roshali De Silva (@RoshaliDe), Hannah Brown (@hannjbrown) and colleagues in the group of Iain Duggin (@iduggin), collaborating with Mecky Pohlschröder (@pohlschr) at the University of Pennsylvania, USA, provides an improved growth medium to study the weird and wonderful morphological plasticity of the archaeon *Haloferax volcanii* [22]. This microbe can change from a discoid-shaped cell to a classical rod-shaped cell, and populations grown in existing media would often have multiple types. Their new media effectively synchronizes these morphological changes across the population by growth phase, hence now allowing much easier experimental study of this phenomenon. Read more about this interesting microbe and shape changing in this month’s blog.

Finally, I wanted to flag something we have been doing to improve the *Microbiology* web site, which is to have a full description of the research interests of all our editors within our new topics area. As potential authors this should help you see how your article fits into one of more of our topic areas and which handling editor(s) you might suggest looks after your submission. Also, we have started putting together exciting plans for the 75th Anniversary in 2022 with many events planned for our annual conference in Belfast which we are all hoping will be a real live event.

Gavin H. Thomas

Department of Biology, University of York, UK, YO10 5YW.
